# Chlorothalonil induces oxidative stress and reduces enzymatic activities of Na^+^/K^+^-ATPase and acetylcholinesterase in gill tissues of marine bivalves

**DOI:** 10.1371/journal.pone.0214236

**Published:** 2019-04-09

**Authors:** Md. Niamul Haque, Hye-Jin Eom, Sang-Eun Nam, Yun Kyung Shin, Jae-Sung Rhee

**Affiliations:** 1 Department of Marine Science, College of Natural Sciences, Incheon National University, Incheon, South Korea; 2 Research Institute of Basic Sciences, Incheon National University, Incheon, South Korea; 3 Southeast Sea Fisheries Research Institute, National Institute of Fisheries Science, Tongyeong, South Korea; 4 Institute of Green Environmental Research Center, Yeonsugu, Incheon, South Korea; Universidade de Brasilia, BRAZIL

## Abstract

Chlorothalonil is a thiol-reactive antifoulant that disperses widely and has been found in the marine environment. However, there is limited information on the deleterious effects of chlorothalonil in marine mollusks. In this study, we evaluated the effects of chlorothalonil on the gill tissues of the Pacific oyster, *Crassostrea gigas* and the blue mussel, *Mytilus edulis* after exposure to different concentrations of chlorothalonil (0.1, 1, and 10 μg L^−1^) for 96 h. Following exposure to 1 and/or 10 μg L^−1^ of chlorothalonil, malondialdehyde (MDA) levels significantly increased in the gill tissues of *C*. *gigas* and *M*. *edulis* compared to that in the control group at 96 h. Similarly, glutathione (GSH) levels were significantly affected in both bivalves after chlorothalonil exposure. The chlorothalonil treatment caused a significant time- and concentration-dependent increase in the activity of enzymes, such as catalase (CAT), superoxide dismutase (SOD), glutathione peroxidase (GPx), and glutathione reductase (GR), in the antioxidant defense system. Furthermore, 10 μg L^−1^ of chlorothalonil resulted in significant inhibitions in the enzymatic activity of Na^+^/K^+^-ATPase and acetylcholinesterase (AChE). These results suggest that chlorothalonil induces potential oxidative stress and changes in osmoregulation and the cholinergic system in bivalve gill tissues. This information will be a useful reference for the potential toxicity of chlorothalonil in marine bivalves.

## Introduction

Chlorothalonil (2,4,5,6-tetrachloro-isophthalonitrile) was discovered in 1964 as an effective alternative biocide in marine paint products [1, 2]. Antifouling agents are widely used to prevent organisms such as oysters, mussels, clams, barnacles, worms, crabs, shrimps, algae, and hydroids from attaching to artificial surfaces (e.g., buoys, fish cages or ships) [3]. In addition, chlorothalonil is a broad-spectrum fungicide, extensively used in agriculture, that may pollute aquatic environments from direct or indirect processes such as spray drift and surface runoff [1, 4]. Some studies observed that chlorothalonil is acutely toxic to aquatic organisms such as ascidians, bivalves, crabs, and shrimp [5–7]. Besides its well-studied acute toxicity, data indicate that the biocide has been detected in seawater and sediment worldwide, ranging from 0.008 μg L^-1^ (0.031 nM) up to 29.78 μg L^-1^ (108 nM) [6, 8–12]. Biotransformation of chlorothalonil and environmental fate of its metabolites (e.g. 4-hydroxychlorothalonil) have highlighted, as several studies suggested that its metabolites are more stable than their corresponding parent compounds [1, 13, 14]. Potential mode of action of chlorothalonil with its metabolites at the biochemical and physiological levels have been extensively studied on marine animals, such as disruption of mitochondrial metabolism [15], inhibition of enzymatic reaction [16], embryotic toxicity and endocrine modulatory effect [14], oxidative stress [17], gill damage [18], and impairment of immune system [2].

Marine bivalves, including pacific oysters and blue mussels, are highly suitable models for ecotoxicological studies for several reasons: their sedentary mode of life, ease of collection, sensitivity to stress, filter-feeding behavior, worldwide distribution, and susceptibility to the bioaccumulation of contaminants [19, 20]. These characteristics position them in the trophic network of most coastal ecosystems, between primary and secondary consumers. Gills are respiratory organs in bivalves and play a critical role in gaseous exchange and feeding; moreover, they are highly exposed to a wide range of anthropogenic ingredients and contaminants [21]. As gills are involved in the maintenance of homoeostasis, their biochemical and physiological profiles reflect the adverse effects of the environment on the organisms and are therefore widely used as biomarkers [22, 23]. However, the various responses of gill tissue over-exposed to environmental stressors, particularly chlorothalonil fungicide, in terms of defense mechanisms, are poorly documented in bivalves. Because of the increasing application of chlorothalonil, it is largely found in aquatic environments and may have cytotoxic effects on aquatic organisms [24]. Once the bivalve takes up pollutants such as chlorothalonil via their gills, these pollutants go through biotransformation reactions, which can damage cellular macromolecules by accelerating the production of reactive oxygen species (ROS) [25]. Lipid peroxidation creates highly toxic products, such as malondialdehyde (MDA) and 4-hydroxynonenal, resulting in constant threats to cells [26, 27]. Moreover, the process involves a set of chain reactions: (a) initiation, where the lipid radicals are generated (initiators are ROS, such as OH· and HOO·), (b) propagation reactions in which the free radicals are converted, and (c) the termination reaction [28]. Endogenous and exogenous oxidative challenges are widely found in aquatic ecosystem along with development of sophisticated antioxidant systems (enzymatic and non-enzymatic) to regulate oxidative stress in aquatic animals [26, 29]. These non-enzymatic antioxidant systems are predominantly substances with low molecular weights, such as glutathione (GSH) or vitamin C, and are scavengers of reactive compounds [30]. In addition, the enzymatic defense mechanism of living organisms consists of enzymatic antioxidants, such as catalase (CAT), superoxide dismutase (SOD), glutathione peroxidase (GPx), and glutathione reductase (GR) [31]. Superoxide dismutase catalyzes the dismutation of superoxide radicals (O_2_^-^ + O_2_^-^ + 2H → O_2_ + H_2_O_2_), CAT eliminates hydrogen peroxide, GPx detoxifies hydroperoxides, and GR re-generates glutathione to facilitate the glutathione redox cycle [14, 15]. The modulation of enzymatic and non-enzymatic responses has been reported in Pacific oysters, *C*. *gigas* and blue mussels, *M*. *edulis* after exposure to several pesticides and azamethiphos, respectively [32–34].

Adenosine triphosphatases (ATPases) are a class of enzymes that import many of the metabolites necessary for cell metabolism and export toxins, waste, and solutes that can hinder cellular processes. Na^+^/K^+^-ATPases control cation transport across the cell membrane and maintain the cellular potential [35, 36]. Na^+^/K^+^-ATPase plays a crucial role in osmoregulation and is widely used as a bioindicator of cellular activity in toxic environments [37]. Acetylcholinesterase (AChE) is responsible for modulating motor and cognitive functions in synaptic neurons through the neurotransmitter acetylcholine [38]. The famous mechanism of cholinergic action is the inhibition of AChE, which is responsible for terminating the transmission of the nerve impulse. Marine organisms use AChE; thus, AChE activity is extensively used as a biomarker of exposure to neurotoxic agents, such as organophosphorus and pesticides, including chlorothalonil [39, 40].

Gills of bivalves are the primary uptake route of waterborne pollutants. Because chlorothalonil has been detected from seawater [6, 8–12], gills are likely to be impacted by waterborne chlorothalonil. The objective of the present study was to investigate the potential effects of waterborne chlorothalonil in the gill tissues of two economically important marine bivalves, the Pacific oyster, *C*. *gigas* and the blue mussel, *M*. *edulis*, to determine the adverse oxidative effects of chlorothalonil. Furthermore, we tested the potential effects of chlorothalonil on the activity of Na^+^/K^+^-ATPase and AChE to estimate whether this compound has potentially adverse effects on the homeostasis of ionic/osmotic balance and cholinergic status. We assumed that study using two bivalves would elicit bivalve-specific common response or species-specific sensitivity upon chlorothalonil exposure.

## Materials and methods

### Culture and chemical exposure

All the experiments using bivalves were approved by the animal care and use committee of Incheon National University. The Pacific oysters and the blue mussels used in this study were collected from the bivalve aquaculture farm (34°51′30.75″N, 128°21′01.04″E) in Tongyeong, Gyungnam, South Korea. There was no source for anthropogenic contamination near the aquaculture farm, such as port, eutrophication, or industrial waste. Chemicals in bivalve body and seawater have been periodically monitored by the National Institute of Fisheries Science (South Korea), as the bivalves are economically important. Adult oysters (≈ 95.4 ± 16.2 mm in length) and blue mussels (≈ 104.4 ± 14.5 mm in length) were reared in filtered seawater aerated by the automated culture system of the Southeast Sea Fisheries Research Institute of the National Institute of Fisheries Science (Tongyeong, South Korea) for 20 days before experiment [40, 41]. The acclimated conditions of the bivalves were maintained at 30 practical salinity units (psu), 20 °C, and 16:8 h light:dark photoperiod.

Chlorothalonil was purchased from Sigma (Sigma-Aldrich, Inc., St. Louis, MO, USA; purity > 99%) and was dissolved in dimethyl sulfoxide (DMSO; Sigma-Aldrich, Inc.), which was used as a carrier. The two bivalve species were exposed to different concentrations of chlorothalonil (0.1, 1, and 10 μg L^-1^) in 60 L filtered seawater (100 L glass aquaria; ≈ 2 L per animal) at 20 °C with constant aeration for 96 h. The tested concentrations were chosen based on the environmental relevant levels of chlorothalonil and toxicity data from literatures [6, 8–12, 42]. The lowest concentration of chlorothalonil (0.1 μg L^-1^) is close to the measured value in surface water [42]. In our exposure condition, no mortality was detected in both species up to 50 μg L^-1^ for 96 h. In each treatment group, 9 specimens were collected at regular intervals (24, 48, and 96 h) during exposure periods, and dissected for gill tissue preparation. Three bivalve samples were pooled, and three pooled samples for each concentration were individually analyzed as biological replicates. During the experiment, half of the filtered seawater with the equivalent concentration of chlorothalonil was changed every 24 h. They were fed daily with a microalgae suspension (4 × 10^6^ cells mL^−1^ containing *Dunaliella* sp., *Isochrysis* sp. and *Tetraselmis* sp.) during exposure periods.

### Malondialdehyde (MDA) analysis

Gills tissue approximately 43 ± 4.5 mg was sampled from each bivalve. Malondialdehyde was measured based on the method previously reported for oyster (*C*. *gigas*) and mussel (*M*. *edulis*) tissues [40, 43]. Tissues were homogenized in 5 volumes of buffer (20 mM Tris, 150 mM NaCl, 10 mM β-mercaptoethanol, 20 μM leupeptin, 2 μM aprotinin and 100 μM benzamidine) and centrifuged at 30,000 *g* for 30 min (4 °C). The supernatant was denatured for 15 min at 75 °C. Since thiobarbituric acid reactives (TBARs) has been criticized for its reactivity towards other compounds, two blanks were incorporated to reduce potential background interference; 1) the gill sample plus the buffer alone without TBARS and 3) the TBARS plus chlorothalonil without gill sample. Subsequently, the absorbance of TBARs in the supernatant was measured at 535 nm in a Thermo Varioskan Flash spectrophotometer (Thermo Fisher Scientific, Tewksbury, MA, USA) using a malonaldehyde bis (1,1,3,3-Tetramethoxypropane, Sigma-Aldrich, Inc.) standard. The blank values were subtracted from entire readings. The concentrations of the lipid peroxidation compounds were expressed as nM of MDA per g of gill tissue.

### Enzymatic activities of antioxidant defense system

After chlorothalonil exposure at different concentrations, total glutathione (GSH) content and the activity of several enzymes (catalase, CAT; superoxide dismutase, SOD; glutathione peroxidase, GPx; glutathione reductase, GR) were measured according to our previous study, with slight modifications (e.g., buffer volume, employed basic instruments) [40, 41]. Gill tissue was dissected from three pooled bivalve specimens per group and was independently analyzed. Based on the manufacturer’s instructions (i.e., minimum 40 mg per assay), approximately 49 ± 5.2 mg of gill tissue was used for each replicate. All tissues were immediately washed or homogenized in different buffers as follows.

The GSH concentration was determined using the Glutathione Assay Kit (Catalog No. CS0260; Sigma-Aldrich, Inc.). The gill tissues treated with different concentrations of chlorothalonil were carefully washed in 0.9% NaCl and rinsed. Subsequently, the samples were homogenized (Teflon homogenizer) in trichloroacetic acid at a ratio of 1:20 (w/v) and centrifuged at 3,000 *g* for 10 min at 4 °C. Glutathione in the upper aqueous layer was quantified at an absorbance of 420 nm using a spectrophotometer and a comparison with standard curves was generated using GSH equivalents (0, 150, and 350 μM).

The total GST analysis was carried out as described in previous studies [40, 44]. Briefly, the treated gill tissues were homogenized (Teflon homogenizer) in cold buffer [0.25 M sucrose, 10 mM Tris, 1 mM ethylenediaminetetraacetic acid (EDTA), 0.2 mM dithiothreitol (DTT), and 0.1 mM phenylmethylsulfonyl fluoride (PMSF), pH 7.4] at a ratio of 1:4 (w/v), followed by centrifugation at 10,000 *g* for 10 min at 4 °C. The cytosolic fraction containing the enzyme was collected for enzymatic assays with 1-chloro-2,4-dinitrobenzene (CDNB) as the substrate. The enzymatic assay was used to monitor the conjugation of CDNB and GSH and the absorbance was measured at 340 nm at 25 °C. All results are based on the total protein soluble content of the samples determined using a Bradford assay [45]. The activity of the enzymes was normalized based on total protein and represented as a percentage of the control.

The activity of GPx and GR were measured in gill tissues using the Glutathione Peroxidase Cellular Activity Assay (Catalog No. CGP1; Sigma-Aldrich, Inc.) and Glutathione Reductase Assay Kits (Catalog No. GRSA; Sigma-Aldrich, Inc.), respectively. The gill tissues treated by chlorothalonil were homogenized in a cold buffer (50 mM Tris-Cl, 5 mM EDTA, and 1 mM 2-mercaptoethanol, pH 7.5) at a ratio of 1:4 (w/v) with a Teflon homogenizer followed by centrifugation at 10,000 *g* for 10 min at 4 °C. The upper aqueous layer was collected for the enzymatic assay. The GPx and GR were quantified using the Thermo Varioskan Flash spectrophotometer (Thermo Fisher Scientific) at an absorbance of 340 nm at 25 °C.

Superoxide dismutase and CAT activity were measured in gill tissues using the SOD (Catalog No. 19160; Sigma-Aldrich Chemie, Switzerland) and Catalase Assay Kits (Catalog No. CAT100; Sigma-Aldrich, Inc.), respectively. The treated gill tissues were homogenized in a cold buffer (0.25 M sucrose, 0.5% triton X-100, pH 7.5) at a ratio of 1:4 (w/v), followed by centrifugation at 3,000 *g* for 30 min at 4 °C. The upper aqueous layer was collected for the enzymatic assay. Total SOD and CAT were quantified using a Thermo Varioskan Flash spectrophotometer (Thermo Fisher Scientific) at an absorbance of 440 nm and 520 nm, respectively at 25 °C.

### Enzymatic activities of Na^+^/K^+^-ATPase and acetylcholinesterase

The Na^+^/K^+^-ATPase activity was measured by the McCormick method [46], as previously conducted in bivalve [47]. Briefly, the method estimates the difference in the amount of adenosine diphosphate (ADP) produced by the samples between control (no inhibitor added) and inhibition reaction mixtures. Ouabain (Sigma-Aldrich, Inc.) was used as an inhibitor of the reaction. The biocide-treated gill tissue was homogenized (1:5, w/v) using a Teflon homogenizer in ice-cold buffer (150 mM sucrose, 10 mM ethylenediaminetetraacetic acid, 50 mM imidazole, and 11.5 mM sodium deoxycholate) and centrifuged at 5,000 g for 1 min at 4 °C. The upper aqueous layer containing the enzyme was collected for the Na^+^/K^+^-ATPase enzymatic assay. Difference between the production of inorganic phosphate in the buffer and the production of phosphate in buffer without K was quantified. Na^+^/K^+^-ATPase was measured using a spectrophotometer (Thermo Fisher Scientific) at an absorbance of 340 nm at 20 °C. Enzyme activity was finally expressed as μmol ADP mg protein^-1^.

AChE activity was determined using acetylthiocholine iodide (ATCh) and 5,5′-dithiobis (2-nitrobenzoic acid) (DTNB) (Sigma-Aldrich, Inc.) according to our previous bivalve studies [40, 48] with slight modifications (e.g. buffer volume, employed basic instruments) of established Ellman method [49]. The reaction mixture was prepared in 100 mM of potassium phosphate buffer (pH 7.4). The biocide-treated gill tissue was homogenized (1:5, w/v) using a Teflon homogenizer in an ice-cold phosphate buffer (0.1 M, pH 8.0) and centrifuged at 5,000 g for 1 min at 4 °C. The supernatant containing the enzyme was collected for the AChE enzymatic assay. Then, 100 μL of the supernatant was added to 1.3 mL of the phosphate buffer (0.1 M, pH 8.0) in a 3 mL cuvette, and 50 μL of DTNB (0.01 M) and 10 μL of ATCh (0.075 M) were added as a substrate. The total AChE enzymatic activity was measured using ATCh, a blank without acetylthiocholine and a blank without sample for 5 min at an absorbance of 412 nm at 25 °C. The enzymatic activity was normalized to total protein in supernatant and was represented as a percentage of the control. AChE activity was calculated in nmoles of hydrolyzed acetylcholine chloride min^-1^ mg protein^-1^ (extinction coefficient ε_412_ = 13,600 M^-1^ cm^-1^).

### Statistical analysis

The SPSS v. 17.0 (SPSS Inc., Chicago IL, USA) software package was used for statistical analyses. Data were expressed as means ± standard deviation (S.D.). Significant differences between the experimental groups were analyzed using a one-way comparison ANOVA followed by a Tukey’s post-hoc test. Any differences with a probability of *P* < 0.05 were considered significant.

## Results

### Intracellular MDA content

**Fig 1:**
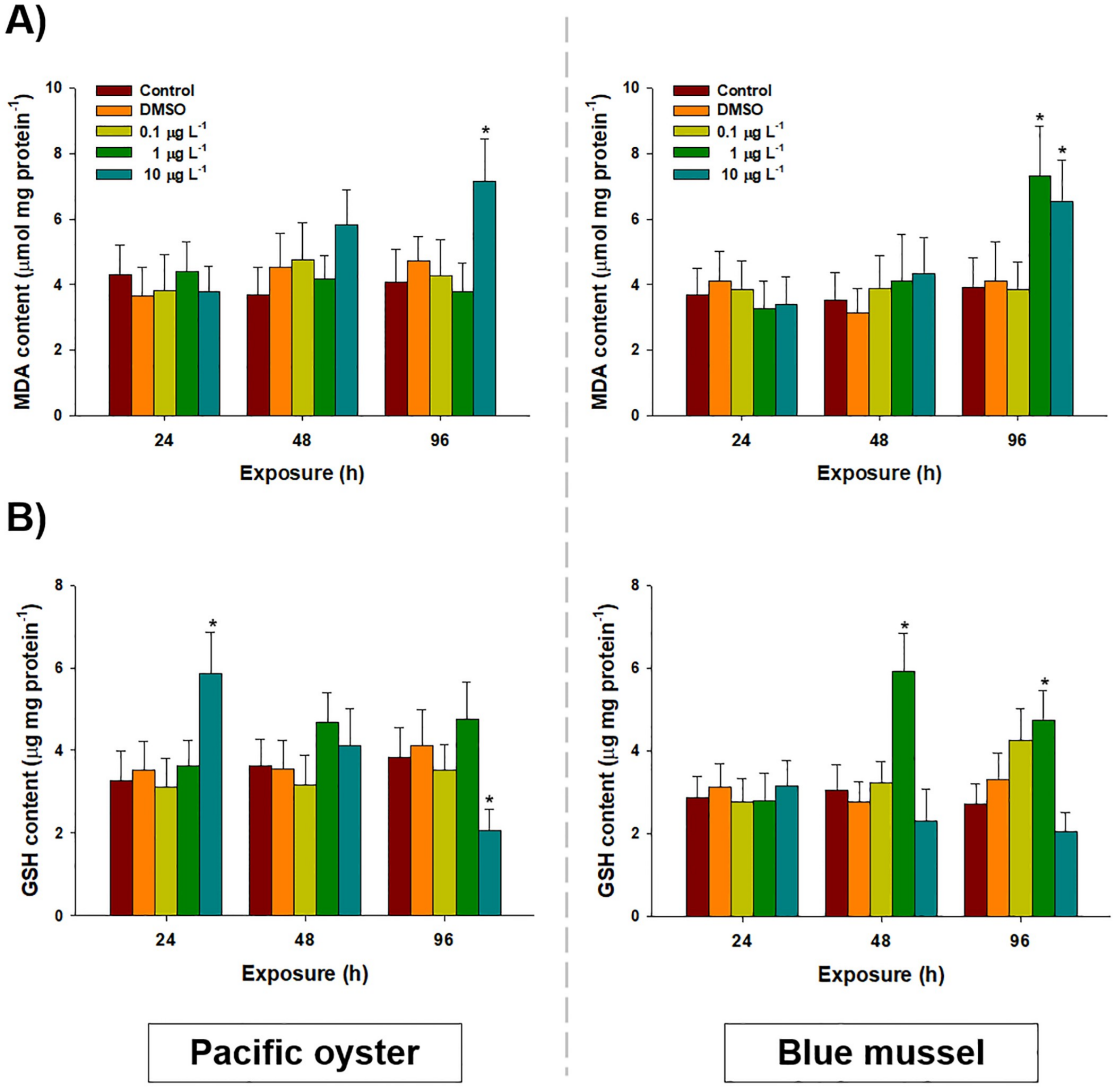
Effects of waterborne chlorothalonil on non-enzymatic oxidative stress parameters of marine bivalves. Effects of different concentrations of chlorothalonil (0.1, 1.0, and 10.0 μg L^-1^) on (A) malondialdehyde (MDA) and (B) glutathione (GSH) levels in the gill tissues of Pacific oyster (*Crassostrea gigas)* and blue mussels (*Mytilus edulis)* for 96 h. Lipid peroxidation measured by TBARS. Data are expressed as the mean ± standard deviation (S.D.). An asterisk (*) indicates significant differences compared to the control (*P* < 0.05).

Overall, MDA levels were increased by waterborne chlorothalonil in the gill tissues of both bivalves (Fig 1A). For *C*. *gigas*, the MDA content was significantly increased at the highest concentration of chlorothalonil (10 μg L^-1^: 7.15 ± 1.29 μM mg^-1^) at 96 h compared to that of the control (4.10 ± 0.97 μM mg^-1^) (*P* < 0.05). In the case of *M*. *edulis*, the MDA level was significantly elevated after exposure to 1 (86%, 7.32 ± 1.52 μM mg^-1^) and 10 μg L^-1^ (66%, 6.52 ± 1.29 μM mg^-1^) of chlorothalonil at 96 h compared to the control (3.93 ± 0.89 μM mg^-1^) (*P* < 0.05). The solvent DMSO and other studied concentrations of chlorothalonil produced no significant alteration in MDA content during the 96-h exposure in both bivalves (*P* > 0.05).

### Intracellular GSH content

Glutathione levels were significantly elevated in the gill tissues of *C*. *gigas* in response to 10 μg L^-1^ of chlorothalonil for 24 h (5.86 ± 1.01 μg mg^-1^) compared to that of the control (3.26 ± 0.72 μg mg^-1^) (*P* < 0.05) (Fig 1B). In addition, the GSH level was significantly depleted after 96 h of exposure to 10 μg L^-1^ of chlorothalonil (2.07 ± 0.51 μg mg^-1^) compared to the control (3.83 ± 0.74 μg mg^-1^) (*P* < 0.05). In the case of *M*. *edulis*, significant increases in GSH levels were observed 48 h (5.93 ± 0.91 μg mg^-1^) and 96 h (4.75 ± 0.71 μg mg^-1^) after exposure to 1 μg L^-1^ of chlorothalonil compared to the control (2.71 ± 0.50 μg mg^-1^) (*P* < 0.05). There was no significant change in GSH after exposure to DMSO and 0.1 μg L^-1^ of chlorothalonil.

### Antioxidant defense system

**Fig 2:**
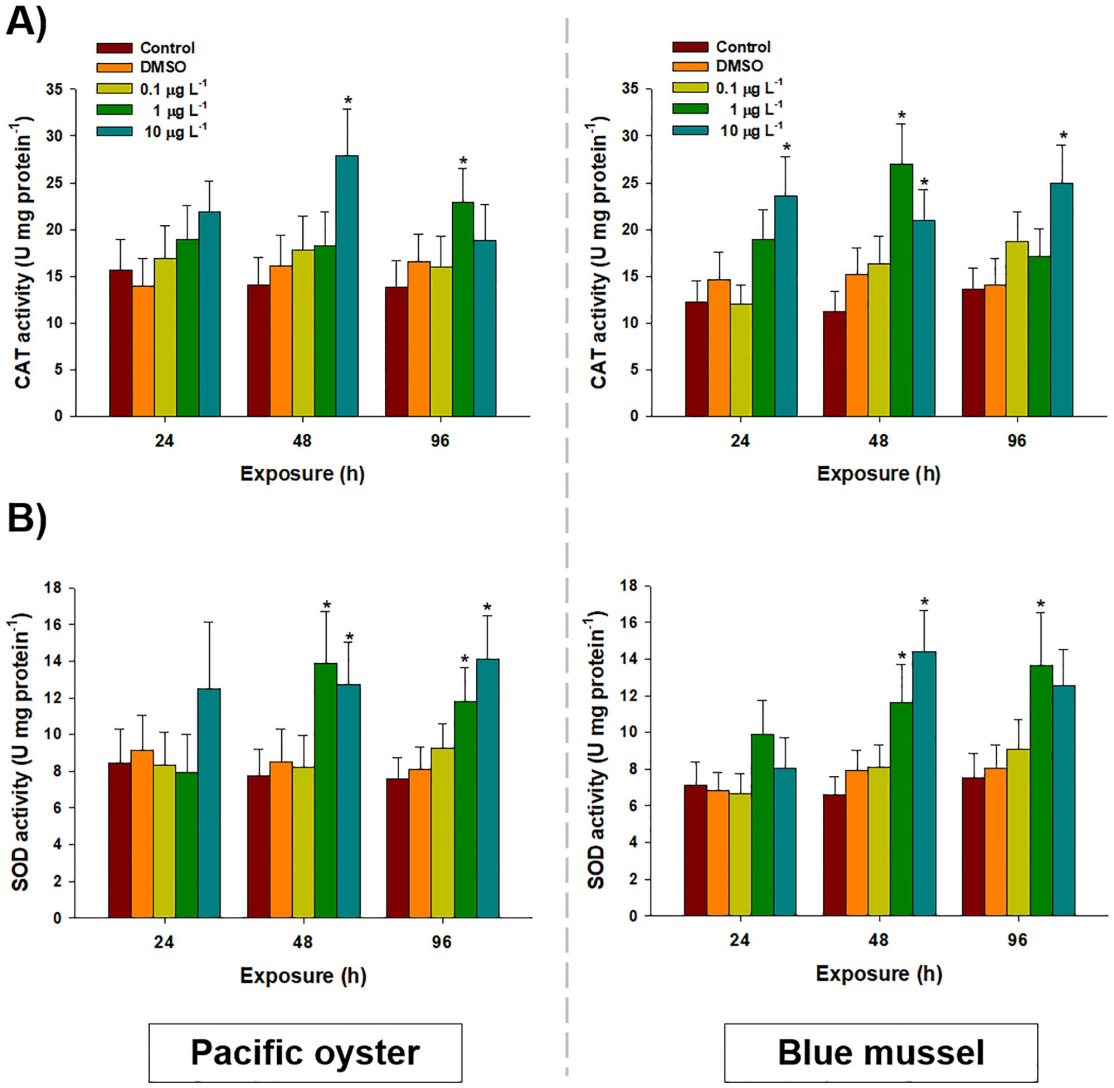
Effects of waterborne chlorothalonil on enzymatic oxidative stress parameters of marine bivalves. Effects of different concentrations of chlorothalonil (0.1, 1.0, and 10.0 μg L^-1^) on (A) catalase (CAT) and (B) superoxide dismutase (SOD) levels in the gill tissues of Pacific oysters (*Crassostrea gigas)* and blue mussels (*Mytilus edulis)* over 96 h. Data are expressed as the mean ± standard deviation (S.D.). An asterisk (*) indicates significant differences compared to the control (*P* < 0.05).

The enzymatic activity of CAT was significantly elevated in the gill tissues of *C*. *gigas* and *M*. *edulis* after exposure to chlorothalonil for 96 h (Fig 2A). Catalase activity was significantly increased after 48 h exposure to 10 μg L^-1^ (27.89 ± 5.01 U mg^-1^; control: 14.12 ± 2.85 U mg^-1^) and 96 h exposure to 1 μg L^-1^ (22.96 ± 3.59 U mg^-1^; control value: 13.86 ± 2.79 U mg^-1^) in the gill tissues of *C*. *gigas* (*P* < 0.05). However, in *M*. *edulis*, CAT activity was significantly increased by 1 μg L^-1^ (26.94 ± 4.36 U mg^-1^) of chlorothalonil compared to the control (11.23±2.11 U mg^-1^) after 48 h (*P* < 0.05). Similarly, significant increases in CAT were detected in *M*. *edulis* at 24 h (23.61 ± 4.21 U mg^-1^), 48 h (21.01 ± 3.28 U mg^-1^), and 96 h (24.93 ± 4.11 U mg^-1^) following exposure to 10 μg L^-1^ of chlorothalonil compared to the control (13.61 ± 2.23 U mg^-1^) (*P* < 0.05).

Superoxide dismutase activity increased in the gill tissues of *C*. *gigas* and *M*. *edulis* after 96 h of exposure to chlorothalonil (Fig 2B). Significantly higher SOD activity was observed in *C*. *gigas* after exposure to 1 μg L^-1^ (13.85 ± 2.84 U mg^-1^) and 10 μg L^-1^ (12.74 ± 2.26 U mg^-1^) of chlorothalonil compared to the control (7.78 ± 1.12 U mg^-1^) (*P* < 0.05). Furthermore, SOD activity was significantly elevated at 96 h after exposure to 1 μg L^-1^ (11.82 ± 1.82 U mg^-1^) and 10 μg L^-1^ (14.11 ± 2.36 U mg^-1^) of chlorothalonil in *C*. *gigas* compared to the control (7.59 ± 1.12 U mg^-1^) (*P* < 0.05). In the case of *M*. *edulis*, significantly increased SOD activity was observed 48 h after exposure to 1 μg L^-1^ (11.61 ± 2.11 U mg^-1^) and 10 μg L^-1^ (119%, 13.39 ± 2.26 U mg^-1^) of chlorothalonil compared to the control (6.58 ± 1.02 U mg^-1^) (*P* < 0.05). A higher level of SOD activity was also measured after 96 h of exposure to 1 μg L^-1^–in *M*. *edulis* (13.67 ± 2.85 U mg^-1^) compared to that in the control (7.52 ± 1.30 U mg^-1^) (*P* < 0.05).

**Fig 3:**
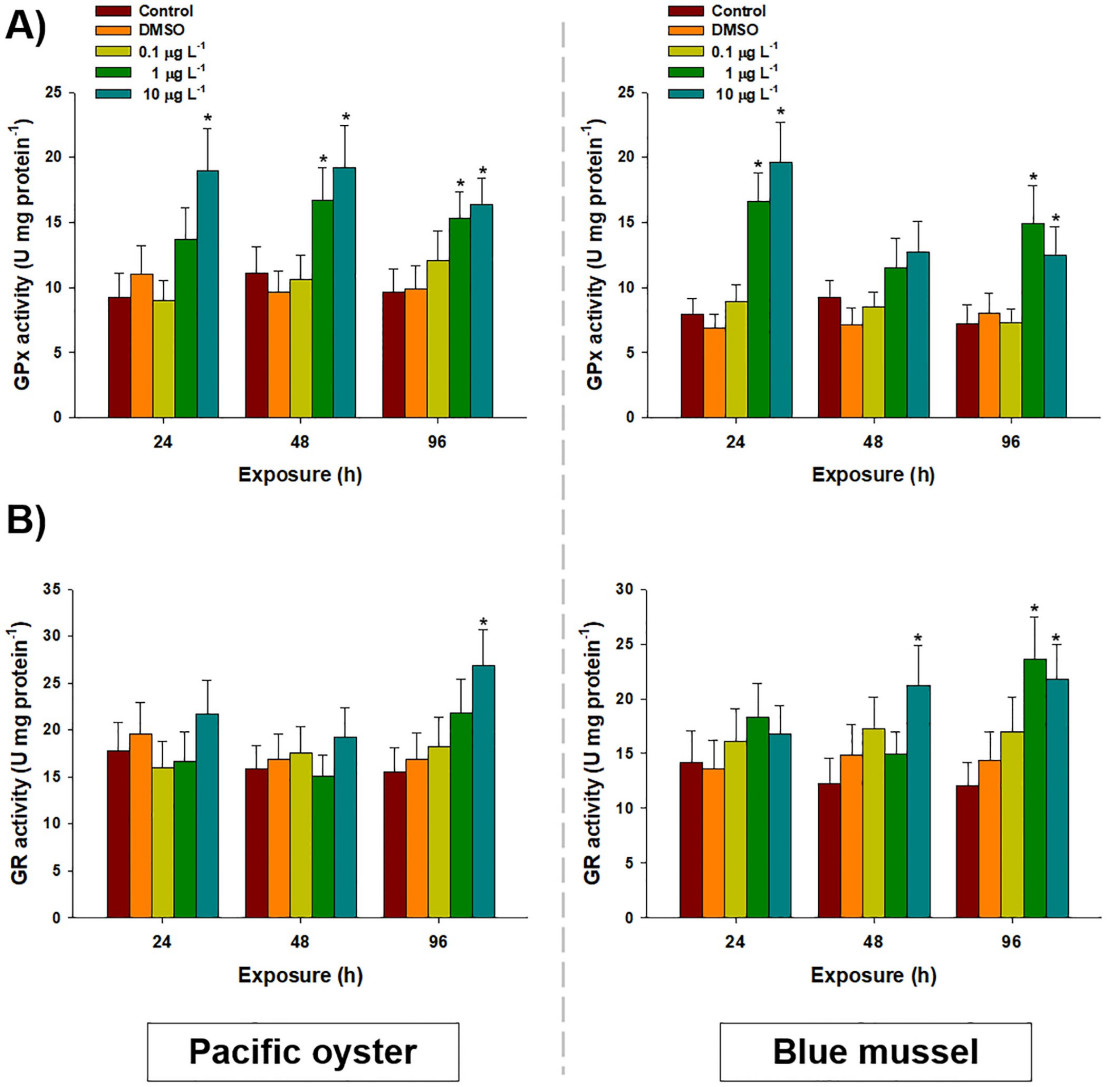
Effects of waterborne chlorothalonil on enzymatic oxidative stress parameters of marine bivalves. Effects of different concentrations of chlorothalonil (0.1, 1.0, and 10.0 μg L^-1^) on (A) glutathione peroxidase (GPx) and (B) glutathione reductase (GR) levels in the gill tissues of Pacific oysters (*Crassostrea gigas)* and blue mussels (*Mytilus edulis)* over 96 h. Data are expressed as the mean ± standard deviation (S.D.). An asterisk (*) indicates significant differences compared to control (*P* < 0.05).

Significant increases in GPx activity was detected in both bivalves exposed to chlorothalonil for 96 h (Fig 3A). In the gill tissue of *C*. *gigas*, GPx activity was significantly increased after exposure to 1 μg L^-1^ of chlorothalonil at 48 h (16.69 ± 2.50 U mg^-1^) and 96 h (15.25 ± 2.01 U mg^-1^) compared to the control (11.16 ± 2.01 U mg^-1^ for 48 h and 9.68 ± 1.77 U mg^-1^ for 96 h). The same was also significantly increased in *C*. *gigas* after exposure to 10 μg L^-1^ of chlorothalonil at 24 h (18.99 ± 3.25 U mg^-1^), 48 h (19.24 ± 3.23 U mg^-1^), and 96 h (16.69 ± 2.02 U mg^-1^) compared to the control (*P* < 0.05). In *M*. *edulis*, GPx activity was significantly increased after 24 h exposure to 1 (16.59 ± 2.23 U mg^-1^) and 10 μg L^-1^ (19.61 ± 3.11 U mg^-1^) of chlorothalonil compared to the control (7.95 ± 1.23 U mg^-1^) (*P* < 0.05). It was also significantly elevated in *M*. *edulis* exposed to 96 h of 1 (14.93 ± 2.86 U mg^-1^) and 10 μg L^-1^ (12.45 ± 2.25 U mg^-1^) of chlorothalonil compared to that in the control (7.25 ± 1.39 U mg^-1^) (*P* < 0.05).

In the gill tissue of *C*. *gigas*, a significant elevation in GR (26.85 ± 3.83 U mg^-1^) was observed in response to exposure to 10 μg L^−1^ of chlorothalonil at 96 h compared to the control (15.55 ± 2.61 U mg^-1^). For *M*. *edulis*, the activity of GR was significantly increased after exposure to 10 μg L^-1^ at 48 h (21.22 ± 3.66 U mg^-1^) and at 96 h (21.77 ± 3.20 U mg^-1^) compared to the control (12.29 ± 2.26 U mg^-1^ for 48 h and 12.06 ± 2.11 U mg^-1^ for 96 h) (*P* < 0.05) (Fig 3B). A significantly higher level of GR activity was also observed at 96 h after exposure to 1 μg L^-1^ in *M*. *edulis* (23.63 ± 3.85 U mg^-1^) compared to the control (12.06 ± 2.11 U mg^-1^) (*P* < 0.05). However, the presence of DMSO, and other studied concentrations of chlorothalonil produced no significant changes in GR content during the 96-h treatment in both bivalves (*P* > 0.05).

### Enzymatic activities of Na^+^/K^+^-ATPase and AChE

**Fig 4:**
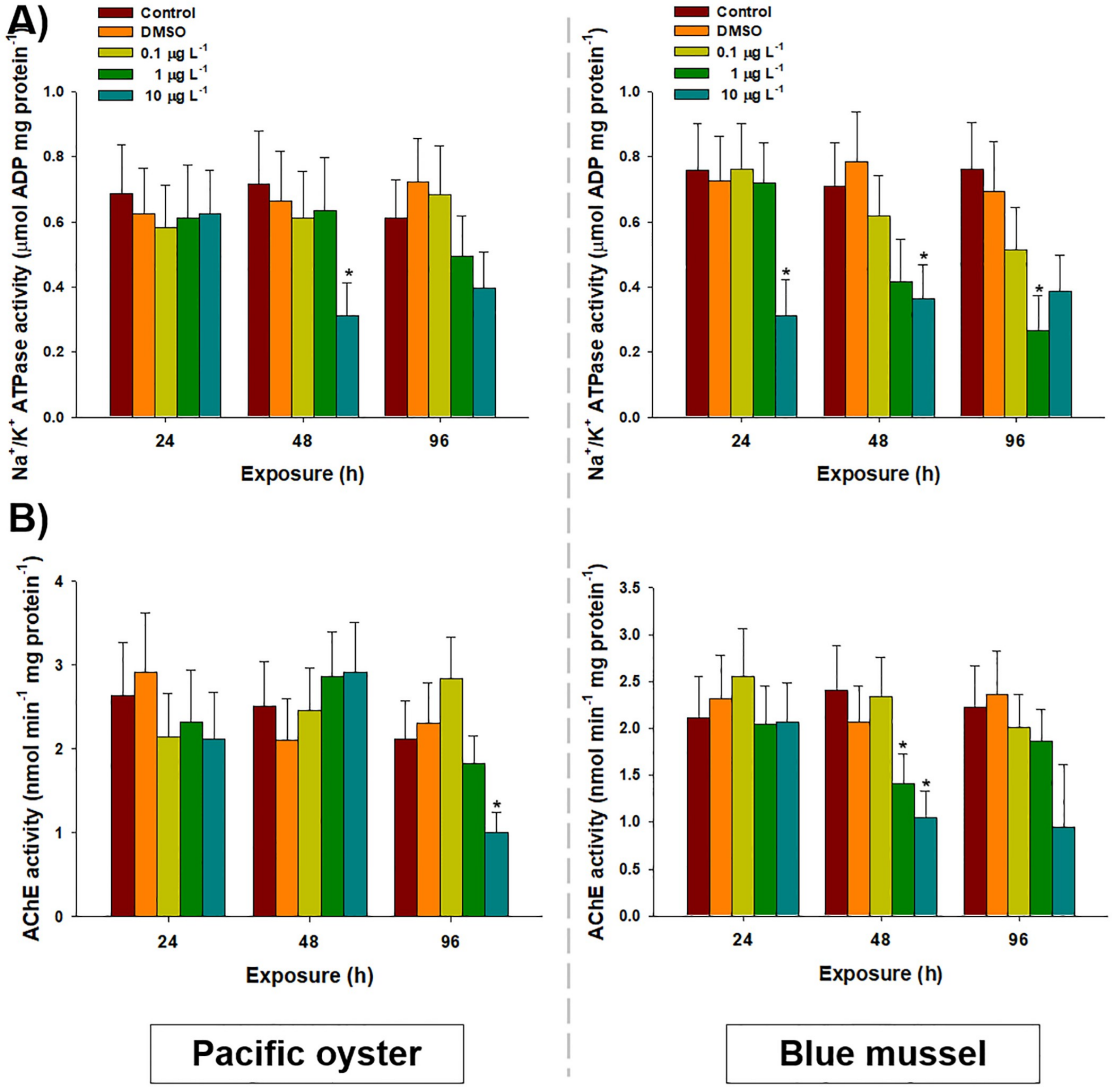
Effects of waterborne chlorothalonil on physiological parameters of marine bivalves. Effects of different concentrations of chlorothalonil (0.1, 1.0, and 10.0 μg L^-1^) on (A) Na^+^/K^+^-ATPase and (B) acetylcholinesterase (AChE) levels in the gill tissues of Pacific oysters (*Crassostrea gigas)* and blue mussels (*Mytilus edulis)* over 96 h. Data are expressed as the mean ± standard deviation (S.D.). An asterisk (*) indicates significant differences compared to the control (*P* < 0.05).

In the gill tissues of *C*. *gigas*, significant decreases in Na^+^/K^+^-ATPase activity were observed at 48 h after exposure to 10 μg L^−1^ (0.31 ± 0.10 μM mg^-1^) of chlorothalonil compared to that in the control (0.71± 0.16 μM mg^-1^) (*P* < 0.05). For *M*. *edulis*, significant decreases in Na^+^/K^+^-ATPase activity were measured at 96 h after exposure to 1 μg L^-1^ (0.27 ± 0.11 μM mg^-1^) of chlorothalonil compared to the control (0.76± 0.14 μM mg^-1^) (*P* < 0.05) (Fig 4A). Significant decreases in Na^+^/K^+^-ATPase activity were also observed at 24 h (0.31 ± 0.11 μM mg^-1^) and at 48 h (0.36 ± 0.11 μM mg^-1^) after exposure to 10 μg L^-1^ of chlorothalonil compared to the control (0.76 ± 0.14 μM mg^-1^ for 24 h and 0.71± 0.13 μM mg^-1^ for 48 h) (*P* < 0.05). No significant changes in Na^+^/K^+^-ATPase activity were detected in the gill tissue of both bivalves exposed to DMSO and 0.1 μg L^-1^ of chlorothalonil (*P* > 0.05).

In *C*. *gigas*, a significantly lower level of AChE was observed at 96 h after exposure to 10 μg L^-1^ (1.01 ± 0.24 nM mg^-1^) of chlorothalonil compared to that in the control (2.11 ± 0.46 nM mg^-1^) (*P* < 0.05). However, in *M*. *edulis*, significant decreases in AChE activity was observed at 48 h after exposure to 1 (1.41 ± 0.32 nM mg^-1^) and 10 μg L^-1^ (1.05 ± 0.28 nM mg^-1^) of chlorothalonil compared to the control (2.41 ± 0.46 nM mg^-1^) (*P* > 0.05) (Fig 4B). However, no significant changes to AChE activity were observed after exposure to DMSO, 0.1, and 1 μg L^-1^ of chlorothalonil at 24 h, 48 h, and 96 h in both bivalves.

## Discussion

Sources of chlorothalonil have been recognized mostly as antifoulants, but they are also useful in agriculture, thus, significantly increasing their abundance in the aquatic environment particularly in coastal areas, subsequently increasing public concern [24, 50]. To date, limited information is available on the consequence of this new-generation of antifoulants on marine invertebrates, particularly in the gill tissues of bivalves. The results obtained in this study suggest that lipid peroxidation in the gill tissues of both bivalves, a toxic effect of chlorothalonil, seems to be time- and concentration-dependent. Significant changes in MDA concentrations were induced in both bivalves at higher chlorothalonil concentrations, however, the gill tissues of *M*. *edulis* were more vulnerable to peroxidation, even at lower concentrations of chlorothalonil (1.0 μgL^-1^), than *C*. *gigas* at 96 h. Stress tolerance in studied bivalves appears higher over short periods of time and at lower concentrations. Our results suggest that chlorothalonil may increase intracellular ROS [24], subsequently inducing lipid peroxidation in gill tissues [51]. In addition, these results suggest that oxidative damage to the cellular membrane, as indicated by increases in MDA levels, may induce an imbalance between the production and the removal or scavenging of oxidants. Similar results of lipid peroxidation in response to organic pollutants comprising antifoulants were observed in other aquatic bivalves such as duck mussels (*Anodonta anatina*) [52], swollen river mussels (*Unio tumidus*) [53], Pacific oysters (*Crassostrea gigas*) [40], and Mediterranean mussels (*Mytilus galloprovincialis*) [54]. Thus, relatively high concentrations of chlorothalonil may induce lipid peroxidation and potential oxidative damage in the gill tissues of marine bivalves.

Exposure to chlorothalonil significantly increased GSH levels in the gill tissues of both bivalves, while the level of GSH depleted significantly in *C*. *gigas* at 96 h after exposure to higher concentrations (10 μg L^-1^). In contrast to the results of past studies on variations in GSH levels between bivalves, *M*. *edulis* has a higher resistance to ROS than *C*. *gigas*. We were not able to incorporate additional important evidences on redox imbalance (e.g. measurement of GSSG and calculation of GSH/GSSG ratio) as our preliminary experiment revealed that their gill cells contain very small amount of GSSG. However, elevations in GSH levels may follow the effects of potential oxidative stress caused by chlorothalonil in the gill tissues of bivalves. The elevated GSH levels suggested the synthesis of new GSH as a free radical scavenger [29]. Previously, it was identified that chlorothalonil is a thiol-reactive compound and is detoxified by conjugation to low molecular weight thiols such as GSH [55–57]. The reason for the depletion of GSH at the highest concentrations in *C*. *gigas* may be attributed to the binding capacity of chlorothalonil with functional cellular groups by nucleophilic substitution reactions through sulfhydryl groups and the detoxification of ROS [58, 59]. Furthermore, the depletion of GSH could reduce the detoxification capacity of the tissues as well as increase their susceptibility to oxidative stress [58]. Previous studies have suggested sensitivities of GSH in bivalves, such as antifouling biocides-exposed *C*. *gigas* [30], t-butyl hydroperoxide-exposed *Rangia cuneata* [60], and contaminants-exposed *C*. *virginica* [61]. Thus, the results demonstrated that GSH elevation may be linked to protection against the potential oxidative stress induced by chlorothalonil. As this explanation needs additional observations of enzymes employed in GSH regulation for clarification, the response of the entire antioxidant defense system was analyzed.

Dose-dependent increases in CAT and SOD in response to chlorothalonil could be interesting indicators of antioxidant defenses and cellular stress, as they are the first line of defense against oxygen toxicity [30, 41]. Strong enzymatic sensitivity of CAT and SOD has been reported in bivalves. For example, the enzymatic activity of CAT increased in *M*. *galloprovincialis* and *Pinna nobilis* exposed to lindane and polluted bay, respectively [62, 63]. The elevation of GPx and GR in the gill tissues of bivalves at higher concentrations of chlorothalonil indicates their strong involvement in regulating GSH metabolism and quenching the flux of reduced oxygen intermediates such as O_2_^−^ and H_2_O_2_ for cellular antioxidant protection [26]. Possible explanations for these results could be that it is an adaptive response of bivalves to the pro-oxidant scenario and to potential adverse effects of chlorothalonil at higher concentrations. Our results were similar to those of *C*. *gigas* exposed to zinc [64] and *M*. *edulis* exposed to benzo[*a*]pyrene and menadione [65]. Thus, increased activity of the antioxidant defense system in response to exposure to chlorothalonil might be due to the capacity of oxygen radicals to overcome the potential imbalance in cellular redox state and to manage cellular homeostasis as well [24]. In addition, species-specific putative hormetic effects promoted by chlorothalonil were observed in specific exposure conditions such as GSH level in the 1.0 μgL^-1^-exposed in blue mussels and catalase activity in the 10 μgL^-1^-exposed Pacific oysters. Since non-lethal stress challenges can trigger hormetic responses that confer improved tolerance to chemical challenges [66–70], further study by employing low concentration of chlorothalonil will be very interesting to understand bivalve-specific hormesis.

To expand our knowledge on the effects of chlorothalonil on ionic homeostasis in bivalve gill tissue, we analyzed the enzymatic response of Na^+^/K^+^-ATPase. Significant decreases in Na^+^/K^+^-ATPase activity were observed in both bivalves at the highest concentrations of chlorothalonil (10 μgL^-1^), while *M*. *edulis* showed sensitivity at the lower concentration (1.0 μgL^-1^) exposure at 96 h. The significant changes in Na^+^/K^+^-ATPase activity were more pronounced in *M*. *edulis* than *C*. *gigas*. This may explain the inability of *M*. *edulis* to counteract the increased passive Na^+^ efflux during chlorothalonil exposure. The significant suppression of Na^+^/K^+^-ATPase enzyme activity might be due to superfluous intracellular oxidative stress induced by the potential toxicity of chlorothalonil, which may cause membrane depolarization and inhibition of neurotransmission [40]. Previous reports on Na^+^/K^+^-ATPase modulation are scarce in bivalves exposed to waterborne chlorothalonil; however, the suppression of Na^+^/K^+^-ATPase by several environmental chemicals has been studied in fish and crustaceans [71, 72]. Thus, the lowered Na^+^/K^+^-ATPase activity might be potentially correlated with chlorothalonil-induced stressful conditions.

Significantly lower AChE activity detected in the chlorothalonil-exposed gill tissues suggests potentially detrimental effects of chlorothalonil on the cholinergic system of bivalves such as impairment of nervous system function. As AChE plays a crucial role in synaptic transmission at cholinergic synapses, the evaluation of AChE activity in gill tissues of mollusk bivalves has been continuously conducted, such as in the analysis of exposure to TBT, pesticides, and heavy metals [40, 73, 74]. To the best of our knowledge, this study is the first to report on the effects of chlorothalonil on AChE activity in the gill tissues of bivalves. Varying sensitivities of AChE activity between bivalves may reflect inherent differences in individual structures and variances in regulatory metabolism for oxidative stress [75]. Similar to the findings of other studies, we assumed that the inhibition of AChE triggered by chlorothalonil may be due to their altered affinity for free-SH groups and significant variations in their function [40, 75].

## Conclusions

In this study, significant increases in MDA levels and the modulation of GSH levels in the gill tissue of bivalves after exposure to chlorothalonil suggests potential oxidative stress. Significant increases in antioxidant defense enzymes could be evidence of the formation of cytotoxic ROS by chlorothalonil. Bivalves seem to have cellular metabolic protections against biocide-triggered potential modulation of redox imbalance. Chlorothalonil-induced suppression of Na^+^/K^+^-ATPase and AChE activity could be used as an indicator of chlorothalonil pollution. As the bivalves are economically important and most aquaculture and fishery systems are located within coastal regions, this study provides information about potential risk of chlorothalonil that may be found in polluted culture areas. The measurements of changes in enzymatic activity to quantify the detrimental effect of chlorothalonil can improve a practical impetus to the area of aquatic toxicology.
